# Supplementary material for: Smuggling on the Nanoscale—Fusogenic Liposomes Enable Efficient RNA-Transfer with Negligible Immune Response In Vitro and In Vivo
Source: Pharmaceutics. 2023 Apr 11;15(4):1210. doi: 10.3390/pharmaceutics15041210 (PMC10146161; doi:10.3390/pharmaceutics15041210)
Supplement: Supplementary file 1 [file pharmaceutics-15-01210-s001.zip › pharmaceutics-2282652-supplementary figures.pdf]

Supplementary Materials:

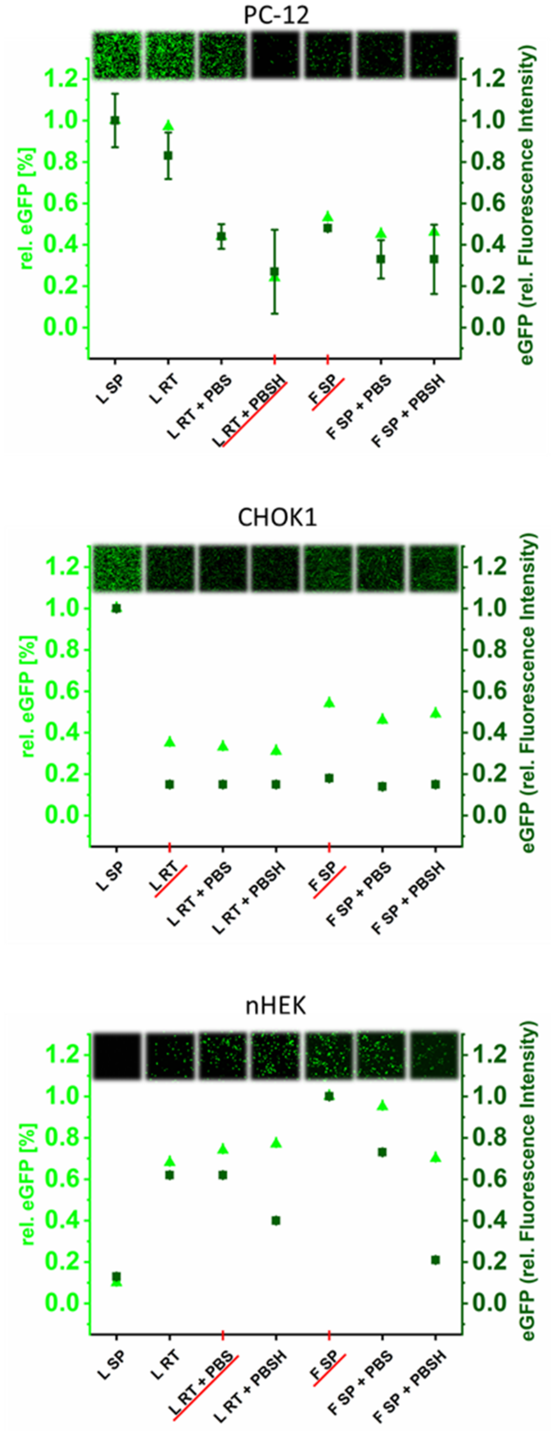

Supplementary Figure S1:
Transfer efficiency and fluorescence intensity after fusion and lipofection: Transfer efficiency (rel. eGFP%) and fluorescence intensity were compared after lipofection (L) and fusion (F) for PC-12, CHOK1, and nHEK cells. The highest intensity and efficiency for each type was set to 1. Different protocols (SP = standard protocol, RT = reduced uptake protocol) and additional washing steps after incubation of lipofection and fusion with PBS and PBS heparin (PBS-H) were used to adjust the transfer quantities of the two transfer reagents for optimal comparability. eGFP-mRNA was used in a concentration of 4 µg/ml for fusion and 2 µg/ml for

lipofection. In all cases a total of 1 µg was transferred per substrate. n = at least 3 independent experiments each.

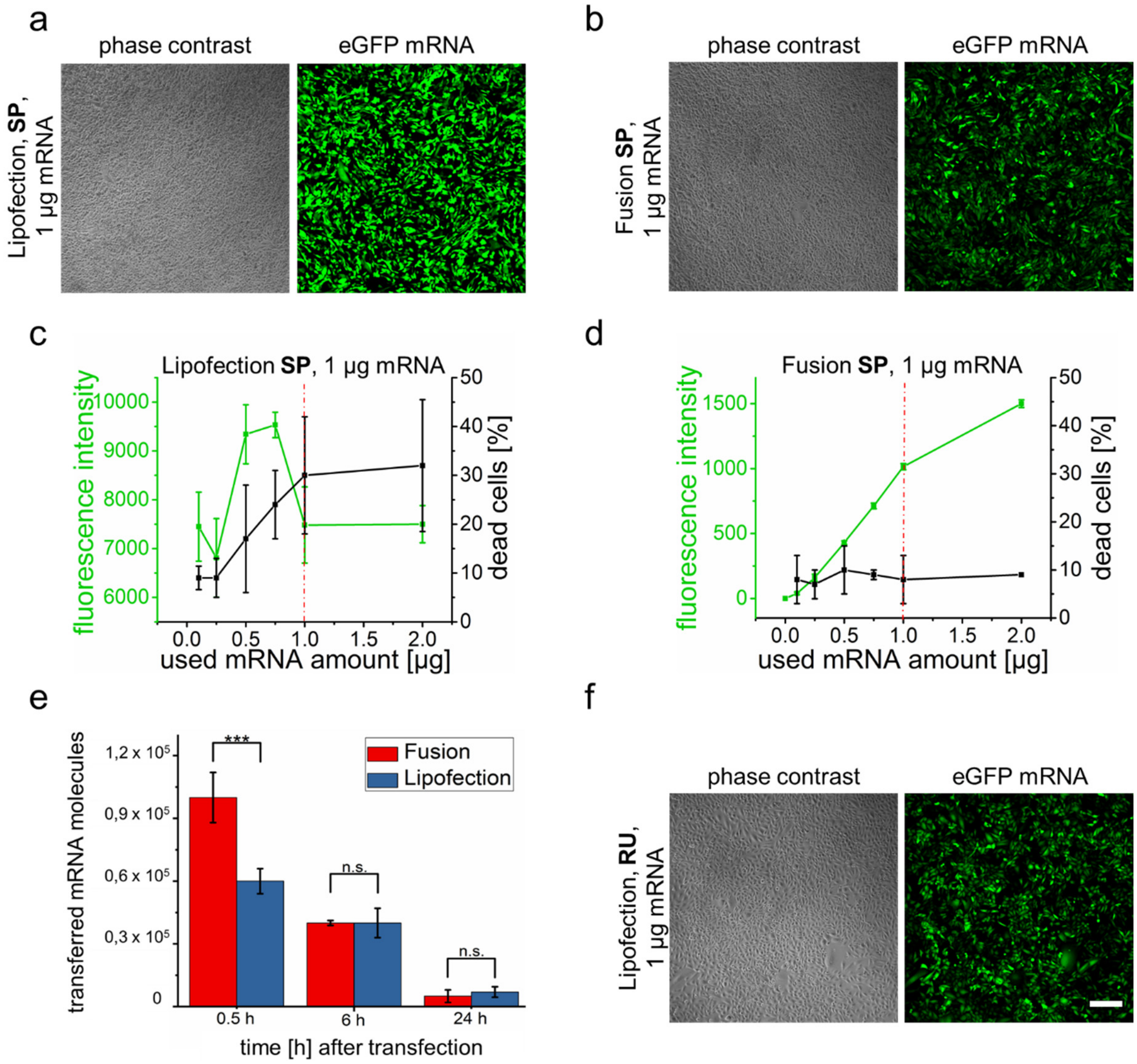

Supplementary Figure S2:

Adjustment of mRNA transfer in CHO-K1 cells by lipofection and fusion. The standard protocols (SP) of lipofection (L) and fusion (F) show significant differences in fluorescence intensity (a+b) by fluorescence microscopy. By varying the amount of mRNA that was used (0.1-2 µg), lipofection does not show a linear increase associated with the increasing amount of mRNA. In contrast, the number of dead cells increases with increasing amounts of mRNA (c). For fusion, there is an almost linear correlation between increasing mRNA amounts used and increasing fluorescence intensities. In addition, the level of dead cells remains unchanged (d). Quantification of the transferred mRNA amounts for L and F by qRT shows that the initially transferred level of mRNA is significantly higher for fusion than for lipofection. In contrast, lipofection shows a more stable mRNA level over time, due to the endocytotic and steady uptake of lipoplexes (e). In f, the adapted, reduced uptake protocol (RU) for lipofection is shown. By adjusting the transferred amount of mRNA with reduced incubation time, better comparability of the systems regarding the influences of the transfer mechanisms on biocompatibility was ensured. eGFP-mRNA was used in a concentration of 4 µg/ml for fusion and 2 µg/ml for lipofection. In all cases a total of 1 µg was transferred per substrate. Scale bar = 200 µm. n = at least 3 independent experiments each.

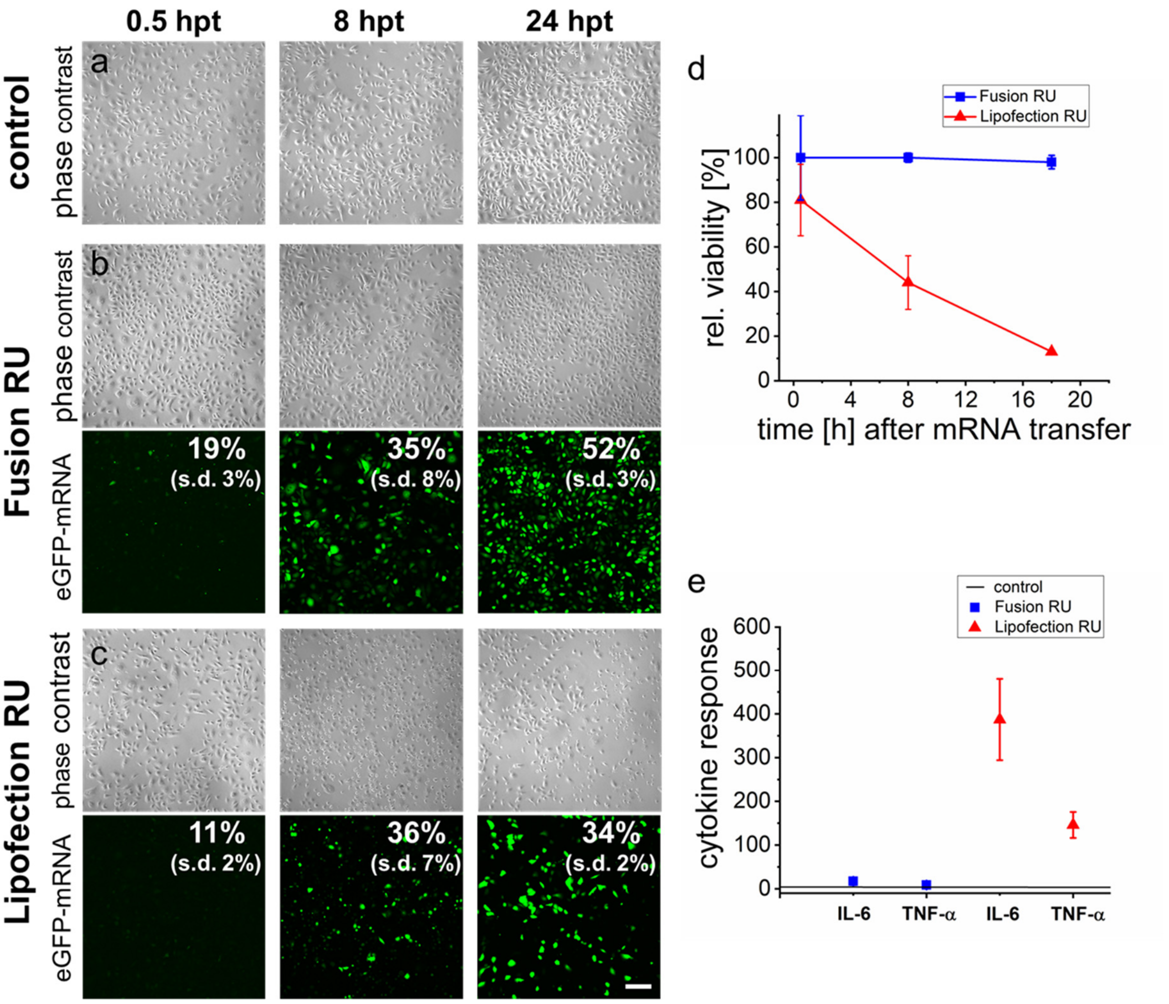

Supplementary Figure S3:

Analysis of cell viability and cytokine response after fusion and lipofection reduced uptake (RU) protocols for nHEK cells. After eGFP-mRNA transfer, expression intensities, cell viability and cytokine responses were analyzed over time for primary nHEK cells as known highly sensitive cell type. Analogous to the analyses of neurons in Figure 2, transfer efficiencies (b+c) cell morphologies (a, b, c) cell viability (d) and cytokine responses (e) of Il-6 and TNF-α have been analyzed after fusion and lipofection. Note the even pronounced cytotoxicity effect in nHEK cells after lipofection while unaffected viability upon direct transfer of mRNA into the cytosol by fusion. eGFP-mRNA was used in a concentration of 4 μg/ml for fusion and 2 μg/ml for lipofection. In all cases a total of 1 μg was transferred per substrate. Scale bar = 200 μm. n = at least 3 independent experiments each.

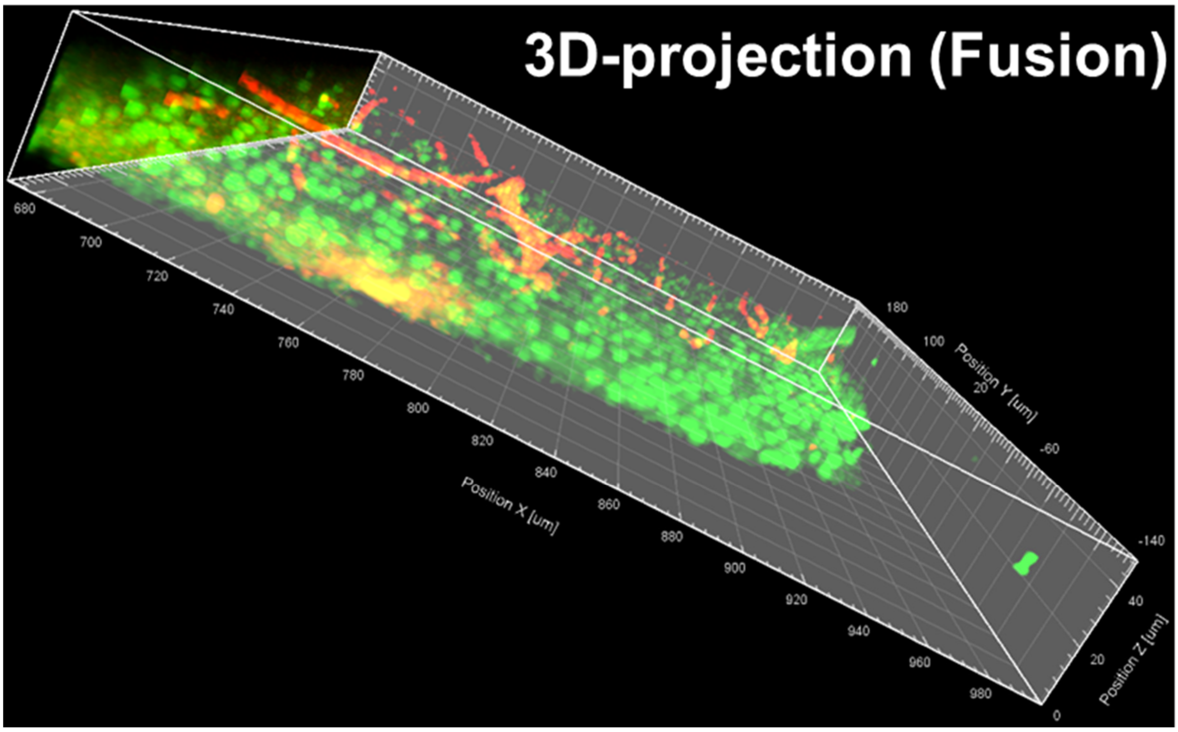

Supplementary Figure S4:
3D projection of the zebrafish telencephalon after fusion-based mRNA transfer. To illustrate the highly efficient transfer of eGFP mRNA into the zebrafish telencephalon, an exemplary section of fusion-based transfer was used to generate a 3D projection with Damaris software. This clearly shows that eGFP can be detected in almost all areas of the telencephalon section. Shown in red is the fluorescent marker dye of FL, which can also be detected in many vessels of the tissue. eGFP-mRNA was used in a concentration of 0.3 ng/µl (corresponds to 1.5 ng per injection).

Supplementary Movie S1

Exemplary imaging of Cal590 analyses for signaling activity of primary neurons. Shown is an exemplary recording of untreated neurons as well as fusion- and lipofection-treated neurons in the absence and presence of endocytosis blocker. Illuminated cells show calcium influx into the neurons and synchronized or activated signal transduction. Detailed analysis is shown in figure 3. eGFP-mRNA was used in a concentration of 4 µg/ml for fusion and 2 µg/ml for lipofection. In all cases a total of 1 µg was transferred per substrate. Scale bar = 200 µm.
